# The fear-increasing *and* fear-decreasing effects of a pilot policy to reduce fear of crime

**DOI:** 10.1371/journal.pone.0282461

**Published:** 2023-03-06

**Authors:** José Miguel Benavente, Daniel Goya

**Affiliations:** 1 Corporación de Fomento de la Producción (Corfo), Chile; 2 Pontificia Universidad Católica de Valparaíso, Valparaíso, Chile; 3 Ministry of Economy, Development and Tourism, Chile; University of Valencia: Universitat de Valencia, SPAIN

## Abstract

Fear of crime has been rising persistently in Chile, even in periods where actual crime rates have decreased, making the perception of crime an important policy issue. This paper presents the results of the impact evaluation of a pilot public policy designed to reduce fear of crime around a shopping centre in Santiago, Chile. The pilot policy consisted of installing a team including police officers and local government officials that handed out information leaflets and talked to passers-by about crime prevention. Pre-intervention and post-intervention surveys were conducted in the shopping centre where the programme was implemented and in a control shopping centre nearby to identify the causal effects of the policy using a difference-in-differences empirical strategy. The results indicate that the programme was effective in reducing fear of crime around the shopping centre, especially at night among its workers, and that it reduced actual crime. However, a deeper analysis suggests that the programme might have actually increased fear of crime among the individuals who directly interacted with the programme. The reduction in crime might have indirectly resulted in an overall reduction in fear among workers, who are likely to be up to date on criminal occurrences in the area, explaining how an increase in fear in those directly contacted is consistent with an overall reduction in fear across workers.

## Introduction

Both being a victim and the fear of being a victim of crime are issues that affect the wellbeing of millions of individuals across the globe [1–3]. In Chile, as in most Latin American countries, the perception of crime is consistently among the top issues of concern among the population according to most opinion polls [4, 5].

As discussed in a review by Hale [6], some of the social costs associated with fear of crime are its psychological effects, changes in individual behaviours, fractures in the sense of community, and increased punitiveness, and fear of crime even risks undermining the legitimacy of the criminal justice system.

There is a wealth of literature on the determinants of fear of crime. There is strong evidence of the roles played by gender and socio-economic factors as determinants of fear. For most variables, however, including age and even previous victimisation, the results do not show a clear effect on fear (see the reviews by Hale [6] and Collins [7] and evidence of possible differences in developing countries in Alda et al. [8]). In public spaces, in particular, there is evidence of greater fear of crime in commercial areas [9], at bus stops [10], and in places in which it would be difficult to escape from potential crime [11].

One theme that is relevant for the interpretation of our findings is the relationship between fear of crime and vicarious victimisation. As argued by [12, 13], previous literature supports the idea that the fear of crime is associated with knowledge about the victimisation suffered by other individuals. Some examples include [14, 15]. There is evidence that the perception of neighbourhood crime trends [16] and spatial proximity to crime [17] affect fear of crime. It is important to understand flows of information about crime, as evidence of a positive relationship between social cohesion and fear of crime is interpreted as being a result of a greater diffusion of information about crimes [18, 19]. This possibility is relevant for the interpretation of our results.

There has been increasing interest in understanding fear of crime in developing countries. See, for instance, [4, 8, 20–22]. In Latin America in particular, there has been work on the determinants of fear of crime [23] and the perceived risk of crime [24] in Peru; the determinants of fear of crime in Brazil and Argentina [25]; the relationship between fear of crime and the media in Paraguay [26]; the effect of fear of crime on trust in the policy and the judiciary in several countries, including Mexico, Argentina and Brazil [27]; and the relationship between fear of crime and social cohesion in Brazil [18, 28]. Another area that has been studied in the region is the relationship between fear of crime and punitive attitudes [29–31].

A topic that has received particular attention in South America is the relationship between fear of crime and public transport, including how it affects behaviour [32–35]. Soto et al. [10] find a higher fear of crime at bus stops, which are public spaces, like the shopping centres where the policy analysed in this paper was implemented.

Since police forces have realised that the usual techniques might not be sufficient to reduce fear of crime [36], community-oriented policing and procedures based on broken windows theory have been the main strategies used to overcome the problem [37]. The former focuses on establishing relationships with the community, while the latter focuses on targeting minor offenses to avoid escalation to more serious forms of crime. Weisburd et al. [38] concluded that broken windows policing does not reduce fear of crime. Evidence for community policing is more encouraging [39, 40] but far from conclusive [41]. The evidence for the effect of police presence on fear is mixed, with some studies indicating that it can reduce fear [39, 42], others indicating that it can be interpreted as being related to higher crime and may thus increase fear [43–45], and some find mixed results [46].

In an effort to try new approaches to deal with the problem of fear of crime, the Chilean Government Lab conducted a pilot intervention in a shopping centre in Santiago, Chile. Developed in a participatory process with the different stakeholders, the intervention consisted of a stand where police officers and local government officials would distribute informative brochures and talk to passers-by about crime prevention.

This paper describes that pilot intervention and presents an impact evaluation of its effects. By doing so, it contributes to knowledge about fear of crime in a commercial context, since most research has been conducted in residential settings, even though it is known that fear of crime can have a negative impact on businesses [47, 48]. The mixed findings provide useful insights for policymakers, but lines of inquiry for further research are suggested to reaffirm some of the conclusions.

## Intervention and data

### Description of the pilot policy

The Chilean *Laboratorio de Gobierno* (Government Lab) was in charge of implementing a pilot policy to reduce fear of crime in the *comuna* (smallest administrative unit) of *Lo Barnechea*, in Santiago, Chile. The first stage of the design of the intervention included local government officials, police officers, and members of the community, and the main conclusion was to focus on the provision of information about crime prevention and hard data on crime in the area. The local government decided to focus the intervention on a shopping centre because according to their knowledge of the area, there were high levels of fear of crime in the surrounding areas, especially among the workers of the shopping centre (most of whome were, according to the local government, residents of the *comuna*).

The intervention consisted of installing a stand where police officers and officials from the local government would talk to passers-by about crime prevention, and distribute brochures with facts about crime in the area and tips on crime prevention. The programme records indicate that the team was present 33 days out of the 95-day period between the first and last interventions (between September and December 2018), for an average of approximately 1.2 hours each time (for a total of 40 hours of intervention). They interacted with approximately 70 individuals per hour on average (for a total of approximately 2,800 total individuals reached directly). There was always at least one person from the local government present (sometimes two), and of the 33 instances of the intervention, there were one or two police officers present during 23 of them.

The rest of the paper describes the data and methodology used to evaluate the impact of the intervention and presents descriptive statistics and results of the evaluation.

### Data

The collection of data to evaluate the intervention was a separate process, independent from the intervention itself. This increases the quality of the intervention and the data gathered, as each team was specialised in its job, and eliminates the conflicts of interest that exists if the data are gathered by the same individuals who are conducting the intervention.

As difference-in-differences was deemed the best method for the evaluation (details in the next section), data before and after the intervention was needed for the shopping centre where the intervention was conducted and for a comparable control zone. Another shopping centre within the same *comuna*, located at a walking distance of seven hundred metres, was used as the control. A pre-intervention survey and a post-intervention survey were conducted in each shopping centre, to passers-by in the same areas where the intervention was conducted. Each round consisted of 200 surveys in each shopping centre, for a total of 800 observations. All the survey data used were completely anonymous.

There is no information on the population of workers and visitors in the shopping centre that would allow us to evaluate how representative the sample is of the population. Nevertheless, any self-selection biases in the respondents should on average be the same across the four surveys (treatment/control and before/after), meaning that the analyses have internal validity. The respondents in the before and after samples were not the same individuals.

The survey was based on the ENUSC (*Encuesta Nacional Urbana de Seguridad Ciudadana*), a large-scale national level survey on crime and related topics that has been conducted yearly since 2005. The survey was simplified relative to the ENUSC, with an emphasis on fear of crime questions but also including the key variables that have been identified by the literature as determinants of fear of crime, to use them as controls. Given the particular interest in fear of crime among workers, the survey included a question about the reason for being at the shopping centre. The post-intervention survey included a question that directly asked whether the individual had interacted with the programme. This question was at the end of the survey to avoid influencing the answers to the other questions.

We do not dig into the debate over how to measure fear of crime [6, 7, 49], given that we decided to measure it in the same way currently used in surveys to measure crime in Chile. The questions used are listed in Table 1. The variables are defined such that a higher value is associated with feeling safer.

**Table 1:** List of fear of crime questions.

Question	Shorthand
According to your experience, how do you feel in the following places?	How safe in general
Walking alone around your neighbourhood.	NH
In shopping centres and their surroundings (in your *comuna*).	SC
In the surroundings of this shopping centre.	This SC
How safe do you feel in the following situations when it is dark?	How safe at night
Walking alone around your neighbourhood.	NH
In shopping centres and their surroundings (in your *comuna*).	SC
In the surroundings of this shopping centre.	This SC

All of the answers were measured on the following four-point scale: 1: very unsafe, 2: slightly unsafe, 3: moderately safe, and 4: very safe.

### Descriptive statistics

Table 2 presents the means for the main variables for three groups: the first two are workers and non-workers in the intervention area, using our survey data (combining the before and after intervention data). As a reference, we present a third column where we use data from the ENUSC 2018 survey to estimate the means for the same variables for the Santiago region. There is significant heterogeneity across *comunas* in Santiago; however, unfortunately, the publicly available ENUSC data identifies the region but not the *comuna* of respondents, so this is the most precise reference we can obtain.

**Table 2:** Descriptive statistics for workers, non-workers, and regional averages.

	Intervention area		Santiago region
	Workers	Non-workers	General population
Age	32.4	34.9	42.98
Woman	0.59	0.54	0.52
Education level of main income provider	6.88	9.39	5.58
Main income provider is employed	0.97	0.94	0.73
Police satisfaction (higher is better)	3.23	3.51	3.19
Victim of robbery (anywhere)	0.24	0.13	0.09
Victim of theft (anywhere)	0.20	0.12	0.06

Estimated mean for each variable. The first two columns present data based on our survey in the intervention area for workers and non-workers. The third column presents estimated means (using expansion factors) for the whole Santiago region from ENUSC 2018 (*comuna* of respondents is not available). The way in which each variable is measured is detailed in S1 Table.

Relative to the means in the region, shopping centre customers (non-workers) are younger, and the main income providers in their households are more educated and more likely to be employed. This is consistent with the sociodemographic profile of residents of *Lo Barnechea*. The workers are younger than both other groups and have a slightly higher education level than the average for Santiago, but still much lower than that of non-workers. There is a higher proportion of women among workers.

The most striking differences are in victimisation: the likelihood of robbery of non-workers in the area is slightly higher than the region’s average, but almost three times as higher for workers in the area than is the average for the region’s population. The differences are starker for theft: non-workers in the area are twice as likely to experience theft than the average resident of the region, and workers in the area are more than three times as likely to experience theft than is the region’s average resident (20% and 6%, respectively). Almost a third of all workers (31%) have suffered from at least one of these forms of victimisation. The fact that the population of the intervention area is not representative of the whole region is not a problem for the causal interpretation of the estimates we present, but it warns against generalising results to other groups.

The perception from the local government that workers of this shopping centre have high levels of fear of crime is consistent with these data on victimisation, assuming a positive relationship between victimisation and fear of crime (although evidence on this is mixed, see [6, 50]).

## Evaluation methods

### Difference-in-differences

The evaluation was designed before the implementation of the programme. A randomised experiment was not feasible. The characteristics of the intervention meant that it was not possible to randomly compel some individuals to interact with it and others to avoid it. Difference-in-differences was chosen as the preferred method to estimate the causal effect of the intervention. This method consists of comparing the change (before and after) in measures of fear of crime in the intervention area, with the change in the same variables in a control area over the same period. The assumption required for this estimator to have a causal interpretation is that the change in the outcome variables in the absence of the programme would have been the same in both areas (this is the ‘parallel trends’ assumption). The credibility of this assumption depends on the quality of the control area, which in this case is highly reliable due to its geographical proximity and socioeconomic similarity. More formally, we can assume that in the absence of the ‘treatment’ (the pilot intervention), the measure of fear of crime for individual *i* in shopping centre *s* at time *t*, *y*_*i*,*s*,*t*_ is determined by the following additive structure:

$$y_{i,s,t}=\lambda_{s}+\tau_{t}+{x_{i,s,t}}^{\prime }\beta +\varepsilon_{i,s,t}$$

where λ_*s*_ and *τ*_*t*_ are place- and time-specific factors, ***x***_***i***,***s***,***t***_ is a vector of individual-level characteristics, and *ε*_*i*,*s*,*t*_ contains the other unobserved, individual-specific determinants of the measure of fear of crime. If the treatment status *treated*_*i*_ (binary variable identifying observations from the shopping centre with the intervention) is unrelated to the change in outcomes, i.e., *E*(*y*_*i*,*s*,1_ − *y*_*i*,*s*,0_|*treated*_*i*_, ***x***_***i***,***s***,***t***_) = *E*(*y*_*i*,*s*,1_ − *y*_*i*,*s*,0_|***x***_***i***,***s***,***t***_), then the difference-in-differences estimate of the impact of the programme for individuals in the shopping centre where the programme was implemented (the ‘average treatment effect on the treated’) corresponds to the *δ* coefficient in the following regression equation:

$$\begin{array}{c} y_{i}={x_{i}}^{\prime }\beta +\gamma_{1}treated_{i}+\gamma_{2}post\_treatment_{i}+\delta treated_{i}\times post\_treatment_{i}+\epsilon_{i} \end{array}$$ (1)

where *treated* is a binary variable that equals one for observations from the treated shopping centre, and *post*_*treatment* is a binary variable equal to one for the post-intervention surveys. The time and shopping centre indices (*t* and *s* respectively) were omitted for simplicity. The individual-level variables in ***x*** are unlikely to cause bias if omitted, but it is useful to include them to increase efficiency. For more details on the methodology, see [51, 52]. The shopping centre which we are using as a control is located nine minutes’ walking distance in the same *comuna* and serves a similar segment of the population.

The variables that are used as controls, which are motivated by the previous literature on fear of crime, are age, gender, having been a victim of theft, having been a victim of robbery, a measure of the opinion of the performance of the police, and a measure of how frequently the individual goes to that shopping centre. In addition, there are three variables related to the household’s main income provider, namely, level of education, a dummy indicating whether he or she is currently employed, and a measure of his or her type of job, included as a fixed-effect given its categorical nature. A measure of household income is not included because the question went unanswered for an important share of respondents. Details on the measurement of all these variables are available in S1 Table.

### Simple differences

The inclusion of a question to identify the individuals who directly interacted with the programme allows for exploring whether the policy had a direct effect on them. However, an assumption much stronger than the ‘parallel trends’ difference-in-differences assumption would be needed to provide these estimates with a causal interpretation. For individuals in the intervention centre in the post-intervention period, if the variable *interacted*_*i*_ identifies the individuals who had a direct relationship with the programme, we can assume that fear of crime is determined as follows:

$$\begin{array}{c} y_{i}=\alpha +{x_{i}}^{\prime }\beta +\delta^{\prime }interacted_{i}+u_{i} \end{array}$$ (2)

If *E*(*u*_*i*_|*interacted*_*i*_, ***x***_***i***_) = *E*(*u*_*i*_|***x***_***i***_), then *δ*′ would estimate the causal effect of the policy on the individuals who interacted with it. However, this assumption is strong, it implies that once the observable characteristics ***x***_***i***_ are controlled for, there are no unobservable differences between the individuals who did and did not interact with the programme. Since interaction with the programme required the individual to approach the programme team, there could be self-selection issues. The exogeneity assumption breaks down, for example, if the individuals who are more fearful of crime are more likely to approach a police officer.

Simple difference estimates are unlikely to be unbiased estimates of causal effects, but we provide evidence that individuals who did and did not interact with the programme are on average almost indistinguishable across a broad set of observable characteristics, suggesting that they are also similar across unobservables. That fact, together with a relatively good set of controls, including measures of victimisation and the individual’s evaluation of the police, suggest that the simple difference estimates still provide useful information.

The methods explained above will be used to explore a possible effect of the programme not only on fear of crime but also on other relevant variables, such as the perception of police effectiveness and actual crime, to inform our interpretation of the results.

All of the calculations and estimations of regression models were conducted using Stata/MP version 15.1.

## Results

### Main result: Causal effect on fear of crime

Table 3 presents the results for the main evaluation of the programme using difference-in-differences. The dependent variables are those listed in Table 1. Only the coefficients for the impact of the policy (*δ* in Eq 1) are reported, for three different samples (the full regression output for these and all of the other regressions reported are available in S1 Appendix).

**Table 3:** Difference-in-differences estimates of the effect on fear of crime.

	How safe in general			How safe at night		
	NH	SC	This SC	NH	SC	This SC
Full sample	-0.0704	0.0821	0.287***	-0.160	0.00681	0.273**
	(0.579)	(0.457)	(0.008)	(0.236)	(0.954)	(0.020)
*N*	769	766	763	767	768	767
Non-workers	0.0228	0.197	0.0830	-0.115	0.0829	-0.0319
	(0.889)	(0.140)	(0.562)	(0.537)	(0.598)	(0.842)
*N*	516	513	512	515	514	515
Workers	-0.0341	0.116	0.0682	0.178	0.0229	0.506*
	(0.915)	(0.695)	(0.825)	(0.567)	(0.943)	(0.097)
*N*	253	253	251	252	254	252

*Notes*. OLS regressions of the difference-in-differences model from Eq 1, using individual-level data from the pre-intervention and post-intervention surveys at the treated and control shopping centres. The dependent variables are those listed in Table 1. Only the coefficient for the difference-in-differences effect of the intervention *δ* is reported, and complete regression output is available in S1 Appendix. The covariates included are age, gender, having been a victim of theft, having been a victim of robbery, a measure of the opinon of the performance of the police, a measure of how frequently the individual goes to that shopping centre; and for the household’s main income provider, his or her level of education, a dummy indicating whether he or she is currently employed, and a measure of his or her type of job (included as a fixed-effect). Robust standard errors. p-values in parentheses.

* p <0.1,

** p <0.05,

*** p <0.01.

The first row includes the whole sample, the second row includes only non-workers at the shopping centres, and the third row includes only workers at the shopping centres. Examining these groups separately is justified not only because of the policy interest in workers. As argued by Dammert and Salazar Tobar [5], the feeling of insecurity in Latin American countries can also be related to deep social changes that have resulted in increased socioeconomic inequalities. Workers and customers at this shopping centre may face different socioeconomic conditions, and as a result, their relationships to crime, the police, and fear of crime might differ.

Examining the full sample first, only the coefficients for ‘walking around *this* shopping centre’ are significant and positive, indicating that the policy had the expected fear-reduction effect. However, this effect is spatially limited to the shopping centre where the programme was implemented. When splitting the sample into workers and non-workers, an interesting result appears: fear-reduction effect at night is driven exclusively by the workers at the shopping centre. The coefficient of 0.5 indicates that the programme, on average, reduced fear by half a point of a four-point scale, or in other words, half of the workers at the shopping centre had a one-point reduction in their level of fear using the four-point scale, which is an important effect.

### Additional results

#### Interacting with the programme

Since the respondents of the post-intervention survey were asked specifically if they had interacted with the programme, we can estimate the relationship between directly interacting with the programme and fear of crime. First, as explained in the methodology section, it is necessary to check whether the individuals who interacted with the programme are similar at least in observable characteristics to those who did not. Tables 4 and 5 show the average values for our control variables for individuals who did and did not interact with the programme, together with the p-values for a two-tailed t-test for differences in means. Table 4 shows results for the whole sample, and Table 5 shows the results for non-workers and workers separately.

**Table 4:** Differences in observable characteristics according to interaction with the programme.

	Whole sample		
	Average value		
	No interaction	Interaction	p-value difference
Age	33.09	40.00	0.023
Woman	0.53	0.46	0.504
Victim of robbery (anywhere)	0.09	0.21	0.124
Victim of theft (anywhere)	0.11	0.14	0.592
Frequency of visits (lower is more frequent)	2.51	2.39	0.579
Education level of main income provider	9.31	9.68	0.235
Main income provider is employed	0.96	0.93	0.994
Police satisfaction (higher is better)	3.46	3.61	0.399

Estimated means for each control, for individuals who did not interact with the programme and individuals who did interact with the programme. The table presents the p-values for a two-tailed t-test of the difference in means between the groups, assuming unequal variances. The way in which each variable is measured is detailed in S1 Table. Additional statistical tests for categorical variables and sample sizes for each group are available in S2 Appendix. The variable ‘type of job’ is excluded because of its qualitative nature, but results with alternative statistical tests (no evidence of significant differences) are available in S2 Appendix.

**Table 5:** Differences in observable characteristics according to interaction with the programme.

	Non-workers			Workers		
	Average value			Average value		
	No interaction	Interaction	p-value difference	No interaction	Interaction	p-value difference
Socioeconomic group	-1.30	-1.38	0.496	-1.91	-2.00	0.837
Age	33.38	39.46	0.070	31.07	43.25	0.144
Woman	0.51	0.46	0.615	0.66	0.50	0.626
Victim of robbery (anywhere)	0.08	0.21	0.148	0.14	0.25	0.684
Victim of theft (anywhere)	0.10	0.13	0.771	0.11	0.25	0.627
Frequency of visits	2.70	2.63	0.729	1.25	1.00	0.033
Education level of main income provider	9.57	9.92	0.084	7.47	8.25	0.690
Main income provider is employed	0.95	0.92	0.532	1	1	-
Police satisfaction (higher is better)	3.50	3.67	0.374	3.18	3.25	0.900

Estimated means for each control, for individuals who did interact with the programme, and for individuals who did interact with the programme. The table presents the p-values for a two-tailed t-test of the difference in means between both groups, assuming unequal variances. The way in which each variable is measured is detailed in S1 Table. Additional statistical tests for categorical variables and sample sizes for each group are available in S2 Appendix. p-values are omitted when it is unfeasible to calculate them. The variable ‘type of job’ is excluded because of its qualitative nature, but results with alternative statistical tests (no evidence of significant differences) are available in S2 Appendix.

For the whole sample, the only significant difference is in age (those who interacted with the programme were on average approximately seven years older). For non-workers, there is a similar difference in ages and a slight difference in the education of the household’s main income provider. For workers, there are differences only in the frequency of visits (with more frequent visits by the workers who interacted with the programme). Given the qualitative nature of most variables, additional results for the Mann-Whitney-Wilcoxon rank-sum test and Pearson’s chi-squared test are reported in S2 Appendix. The only difference from the results from Tables 4 and 5 is some evidence of differences in the reported rates of robbery, which are significantly higher for those who interacted with the programme but only across non-workers. Crime victims (among non-workers) were more likely to interact with the programme than non-crime victims; if this is indicative of unobservables that are also related to fear, estimates should not be interpreted causally for non-workers. All of the other tests indicate that the groups are comparable, suggesting that the estimates might be close to a causal effect, especially for workers.

Table 6 has the same structure as Table 3; however, the coefficients are not the effects estimated through difference-in-differences but the simple difference in fear of crime between those who did and did not interact with the programme (*δ*′ in Eq 2), controlling for the same factors as before.

**Table 6:** Estimates of the effect of interacting with the intervention on fear of crime.

	How safe in general			How safe at night		
	NH	SC	This SC	NH	SC	This SC
Full sample	-0.289	-0.289	-0.476**	-0.284	-0.618***	-0.463**
	(0.195)	(0.195)	(0.018)	(0.220)	(0.004)	(0.048)
*N*	176	176	175	176	174	177
Non-workers	-0.0310	-0.451*	-0.247	-0.0943	-0.398*	-0.184
	(0.879)	(0.083)	(0.210)	(0.707)	(0.087)	(0.408)
*N*	151	151	150	151	149	152
Workers	-1.455***	-1.413***	-1.552***	-1.272***	-1.784***	-1.689***
	(0.009)	(0.001)	(0.003)	(0.002)	(0.001)	(0.007)
*N*	25	25	25	25	25	25

*Notes*. OLS regressions of the simple differences model from Eq 2, using individual-level data from the post-intervention survey at the treated shopping centre. The dependent variables are those listed in Table 1. Only the coefficient for the simple differences effect of the intervention *δ*′ is reported, and complete regression output is available in S1 Appendix. The covariates included are age, gender, having been a victim of theft, having been a victim of robbery, a measure of the opinon of the performance of the police, a measure of how frequently the individual goes to that shopping centre; and for the household’s main income provider, his or her level of education, a dummy indicating whether he or she is currently employed, and a measure of his or her type of job (included as a fixed-effect). Robust standard errors. p-values in parentheses.

* p <0.1,

** p <0.05,

*** p <0.01.

The sample sizes are smaller because only observations from the post-intervention survey at the treated shopping centre can be used (and only those without missing information for covariates).

Some coefficients are significant and *negative* for the full sample and for non-workers, and *all coefficients are significant and negative for workers*. The negative coefficients imply that if these estimates have a causal interpretation–which according to Table 5 is more likely for workers–the programme increased fear of crime among those directly treated, which at first sight seems inconsistent with the difference-in-differences estimates. If there are differences in unobservables between the two groups (and thus the estimates are not causal effects), one interpretation could be that workers who were initially more fearful were more likely to approach the intervention. We conduct some additional exercises to help us interpret this result.

#### Police evaluation

The literature suggests that there could be a relationship between confidence in the police and fear of crime [7, 27, 53]. Now, we explore whether there is evidence of an effect of the policy on individuals’ evaluations of the police as a possible mechanism through which the programme could be affecting fear of crime. Table 7 presents difference-in-differences estimates and the simple differences estimates for the impact of interacting with the programme for the three samples. For comparison, it presents the results not only for trust in the police but also for trust in the local government (which helped deliver the programme) and the judiciary (for which the programme should have no effect). All of the estimates are insignificant.

**Table 7:** Estimates of the effect on the evaluation of different institutions.

	Differences-in-differences			Interacted with the intervention		
	Local gov.	Judiciary	Police	Local gov.	Judiciary	Police
Full sample	0.00898	-0.0557	-0.0700	0.00852	0.00279	0.198
	(0.953)	(0.667)	(0.643)	(0.973)	(0.991)	(0.373)
*N*	661	751	774	154	171	178
Non-workers	-0.0553	-0.150	-0.126	0.0123	0.0850	0.295
	(0.774)	(0.365)	(0.521)	(0.965)	(0.766)	(0.206)
*N*	455	504	520	133	146	153
Workers	0.513	0.255	0.0721	0.191	-0.259	-0.290
	(0.214)	(0.465)	(0.841)	(0.799)	(0.583)	(0.765)
*N*	206	247	254	21	25	25

*Notes*. OLS regressions of the difference-in-differences model from Eq 1 (left panel) and the simple differences model from Eq 2 (right panel), using individual-level data from the pre-intervention and postintervention surveys at the treated and control shopping centres. The dependent variables are measures of the evaluation of the different institutions on a five-point scale, with a higher score indicating a better evaluation. Only the coefficients for the effects of the intervention *δ* and *δ*′, are reported, and complete regression output is available in S1 Appendix. The covariates included are age, gender, having been a victim of theft, having been a victim of robbery, a measure of how frequently the individual goes to that shopping centre; and for the household’s main income provider, his or her level of education, a dummy indicating whether he or she is currently employed, and a measure of his or her type of job (included as a fixed-effect). Robust standard errors. p-values in parentheses.

* p <0.1,

** p <0.05,

*** p <0.01.

#### Victimisation

One possible explanation for the seemingly inconsistent results from Tables 3 and 6 is that the direct effect of the programme is an increase in fear of crime but that, at the same time, police presence reduced actual crime. If workers are aware of the crime levels around the shopping centre, a reduction in crime could have resulted indirectly in a reduction in fear of crime among this group.

Table 8 shows in its first column the difference-in-differences estimates for being a victim of any crime and, in the second column, for being a victim of any crime near the shopping centre where the individual was interviewed. The results show a weakly but significant negative effect on crime (reduction in victimisation) but only for crimes occurring near the shopping centre. This result supports the idea that the reason for an overall fear-reduction effect, together with a fear-inducing one among those directly treated, could be that increased police presence reduced crime, which is what translated into overall reduced fear across workers, something we discuss in more detail below.

**Table 8:** Difference-in-differences estimates of the effect on victimisation.

	Victim	Victim near the shopping centre
Full sample	0.0390	-0.0428*
	(0.541)	(0.062)
*N*	786	786
Non-workers	0.0275	-0.00616
	(0.747)	(0.862)
*N*	529	529
Workers	-0.00150	-0.0704
	(0.992)	(0.363)
*N*	257	257

*Notes*. OLS regressions of the difference-in-differences model from Eq 1, using individual-level data from the pre-intervention and post-intervention surveys at the treated and control shopping centres. The dependent variables are those listed in Table 1. Only the coefficient for the difference-in-differences effect of the intervention *δ* is reported, and complete regression output is available in S1 Appendix. The covariates included are age, gender, a measure of the opinon of the performance of the police, a measure of how frequently the individual goes to that shopping centre; and for the household’s main income provider, his or her level of education, a dummy indicating whether he or she is currently employed, and a measure of his or her type of job (included as a fixed-effect). Robust standard errors. p-values in parentheses.

* p <0.1,

** p <0.05,

*** p <0.01.

## Discussion

This paper presented the results of the impact evaluation of a pilot programme that aimed to reduce fear of crime around a shopping centre in Santiago, Chile. The policy consisted of setting up a stand where local government officials and members of the police engaged in conversations with passers-by about crime prevention and distributed brochures with crime prevention tips and facts about crime in the area.

The difference-in-differences estimates showed an overall fear-reduction effect across workers, spatially circumscribed around the area close to the shopping centre where the programme was implemented. Using information about who interacted directly with the programme, it is possible to explore whether interacting with the officials had an impact on the individual level. There is a significant relationship between interacting with the programme and higher fear of crime. There are two possible explanations for this outcome: it could reflect that more fearful individuals are more likely to approach the police or that interacting with the police increased their fear. The similarity across individuals who did and did not interact with the programme–especially across workers–even in variables such as police satisfaction, together with previous evidence of a possible increase in fear associated with police presence [43, 44], suggests that the latter explanation is feasible.

How can we reconcile the fear-reduction effect obtained for workers of the ‘treated’ shopping centre with the fear-induction effect observed across those who directly interacted with the programme? The results also show that the policy had the effect of reducing *actual crime* in the intervened shopping centre. There could be three mechanisms at play. First, the direct effect of the programme (i.e., on those who approached it) was negative: fear increased, a result consistent with [43–46]. [43, 44]. Second, police presence resulted in a reduction in crime rates. This outcome is not surprising considering the evidence of the crime-deterring effect of police presence [54–57]. Third, information about the lower crime rates was transmitted through informal interactions between the workers at the shopping centre, leading to an overall reduction in fear of crime among workers at the intervened shopping centre. This outcome is consistent with the strong evidence of the effect of vicarious victimisation on fear of crime [6, 13]. Specifically, [16] shows evidence that fear of crime can be affected by the perception of neighbourhood crime trends, and [17] shows that spatial proximity to crime has an effect on fear. Moreover, workers as a group probably have a certain degree of social cohesion, which could lead to higher transmission of information about crime and thus a relationship between effective crime and fear (see the discussions in [18, 19]).

The evidence for the second and third mechanisms described above is stronger, based on difference-in-differences causal estimates. The evidence that the first mechanism (the fear-increasing direct effect of interacting) is a causal impact of the intervention is weaker, but even if it is not, that would not affect the rest of the interpretation, i.e., the intervention reduced fear through a reduction in overall crime in the area, but the effect was only on workers, who are up to date on changes in the victimisation levels in the area.

As argued by Bennett [58] and Abbott et al. [40], police presence or community-oriented practices might not directly reduce fear but might require a simultaneous reduction in crime or perceived disorder to have that effect, which could be what we are observing here. The potential of there being a fear-inducing effect only when there is no reduction in actual crime rates should be considered when designing policies to reduce fear of crime that involve interaction with the police.

The descriptive statistics showed that the population of workers and non-workers of the shopping centre where the intervention was conducted is not comparable to the average resident of the Santiago region. This is not a concern for the causal interpretation of our results (which hinges on the similarity between treatment and control areas), but it suggests caution when generalising the findings to other contexts.

## Conclusions and further research

Our results show that attempts to reduce fear of crime based on local goverment officials and police officers talking to individuals about crime prevention can have mixed results. Our results suggest that interacting with police officers could actually increase individuals’ fear of crime. However, we also have strong evidence that the intervention in this study reduced fear of crime across workers of the shopping centre in general and that it reduced actual crime. It is possible that this reduction in effective crime was what ended up reducing fear. However, lingering issues imply that further research is needed.

The reduction occurred only among workers (who were actually the main target group). One possible explanation is that this is a relatively small and stable group through which updated information about crime is easily shared, something that cannot be scaled up to other contexts. Confirming the role of the reduction in crime on the effect on fear, and the mechanisms through which it occurred requires further research.

For two of the estimated effects (the reduction in crime and the reduction in fear of crime across workers), the results are highly reliable, but the estimated fear-increasing effect of directly interacting with the intervention at the individual level relies on stronger assumptions. The impossibility of estimating credible causal effects of the direct interaction with the intervention is a limitation of our work that should be addressed in future research.

A third consideration is that the workers, and especially the non-workers visiting the shopping centre where the intervention was conducted, are not representative of the overall population. While this is not a problem for the internal validity of the estimates, it limits their external validty (i.e., the possibility of generalising the findings to other groups). Similar policy experiments in different socioeconomic contexts would be useful to reaffirm the findings.

## Supporting information

S1 Data Full dataset used in .dta format. (ZIP)

S1 Table List of variables. List of variables used and how they are measured.
(PDF)

S1 Appendix Detailed regression output. The detailed output of all of the regressions reported in the paper.
(PDF)

S2 Appendix Additional balancing tests. Extensions of Tables 4 and 5 with statistical tests for discrete variables.
(PDF)
