# Supplementary material for: The fear-increasing and fear-decreasing effects of a pilot policy to reduce fear of crime
Source: PLoS One. 2023 Mar 6;18(3):e0282461. doi: 10.1371/journal.pone.0282461 (PMC9987788; doi:10.1371/journal.pone.0282461)
Supplement: S1 Table — List of variables used and how they are measured. (PDF) [file pone.0282461.s002.pdf]

S1 Table

Table 1: **Measurement of variables**

**Variable**	**Name in regression**	**Measurement**
According to your experience, how do you feel in the following places?	How safe in general	1: Very unsafe, 2: Somewhat unsafe, 3: Relatively safe, 4: Very safe
How safe do you feel in the following situations when it's dark?	How safe at night	1: Very unsafe, 2: Somewhat unsafe, 3: Relatively safe, 4: Very safe
Age	Age	Age in years
Woman	Woman	Binary, 1 for women
Victim of robbery (anywhere)	Victim of robbery	Binary, 1 for victim
Victim of theft (anywhere)	Victim of theft	Binary, 1 for victim
Victim of any crime (anywhere)	Victim (as outcome)	Binary, 1 for victim
Victim of any crime (near the shopping centre)	Victim near the shopping centre (as outcome)	Binary, 1 for victim
Frequency of visits to this shopping centre	Frequency of visits	1: Three or more times a week, 2: One or two times a week, 3: At least once a month, 4: At least once every three months, 5: Less than once every three months
Education level of main income provider	Education of income provider	Education levels ranging from 1 (incomplete primary or less) to 11 (postgraduate degree)
Main income provider is employed	Income provider is working	Binary, 1 for working
Type of job of main income provider	Income provider occupation FE (fixed effects)	Nine different categories
Satisfaction with the police	Police satisfaction (as control) / Police (as outcome)	1: Very low, 2: Low, 3: Regular, 4: High, 5: Very high
Satisfaction with local government	Local gov.	1: Very low, 2: Low, 3: Regular, 4: High, 5: Very high
Satisfaction with judiciary	Judiciary	1: Very low, 2: Low, 3: Regular, 4: High, 5: Very high
