# Supplementary material for: The fear-increasing and fear-decreasing effects of a pilot policy to reduce fear of crime
Source: PLoS One. 2023 Mar 6;18(3):e0282461. doi: 10.1371/journal.pone.0282461 (PMC9987788; doi:10.1371/journal.pone.0282461)
Supplement: S1 Appendix — The detailed output of all of the regressions reported in the paper. (PDF) [file pone.0282461.s003.pdf]

S1 Appendix

Table 2: Table 2 details, full sample

	How safe in general			How safe at night		
	NH	SC	This SC	NH	SC	This SC
Treated x Post	-0.0704	0.0821	0.287***	-0.160	0.00681	0.273**
	(0.579)	(0.457)	(0.008)	(0.236)	(0.954)	(0.020)
Post intervention	0.226**	0.0303	-0.139	0.208**	0.212**	-0.0506
	(0.032)	(0.721)	(0.100)	(0.050)	(0.025)	(0.595)
Treated	0.161*	0.00331	-0.266***	0.256**	0.0403	-0.228***
	(0.100)	(0.967)	(0.001)	(0.014)	(0.631)	(0.005)
Age	-0.00329	-0.00402*	-0.00410**	-0.000624	-0.000219	-0.00172
	(0.175)	(0.053)	(0.048)	(0.811)	(0.918)	(0.432)
Woman	-0.339***	-0.214***	-0.262***	-0.336***	-0.237***	-0.338***
	(0.000)	(0.000)	(0.000)	(0.000)	(0.000)	(0.000)
Victim of robbery	-0.0829	-0.0642	-0.188**	-0.108	-0.143	-0.194**
	(0.448)	(0.508)	(0.041)	(0.321)	(0.159)	(0.046)
Victim of theft	-0.186*	-0.116	-0.212**	-0.161	-0.0401	-0.191**
	(0.098)	(0.224)	(0.021)	(0.133)	(0.688)	(0.040)
Police satisfaction	0.0666**	0.103***	0.0838***	0.0611*	0.0798**	0.0771**
	(0.050)	(0.000)	(0.002)	(0.093)	(0.015)	(0.015)
Frequency of visits	-0.0106	0.0166	0.0294	-0.0168	-0.0148	-0.0156
	(0.714)	(0.487)	(0.233)	(0.593)	(0.591)	(0.572)
Education of income provider	0.0257	0.0279*	0.0404***	0.0133	0.00691	0.0218
	(0.185)	(0.088)	(0.010)	(0.516)	(0.693)	(0.168)
Income provider is working	-0.247	0.489	0.514	-0.245	-0.105	-0.826***
	(0.570)	(0.193)	(0.183)	(0.418)	(0.724)	(0.003)
Income provider occupation FE	✓	✓	✓	✓	✓	✓
Sample size	769	766	763	767	768	767
R2	0.145	0.114	0.120	0.102	0.091	0.113

See details in the main text. Income provider refers to the main income provider of the respondent's household. Robust standard errors. p-values in parentheses. * p <0.1, ** p <0.05, *** p <0.01.

Table 3: Table 2 details, non-workers

	How safe in general			How safe at night		
	NH	SC	This SC	NH	SC	This SC
Treated x Post	0.0228	0.197	0.0830	-0.115	0.0829	-0.0319
	(0.889)	(0.140)	(0.562)	(0.537)	(0.598)	(0.842)
Post intervention	0.143	-0.103	0.0859	0.122	0.126	0.219
	(0.306)	(0.314)	(0.483)	(0.425)	(0.333)	(0.113)
Treated	0.0602	-0.114	-0.0557	0.161	-0.0481	0.0314
	(0.666)	(0.285)	(0.635)	(0.318)	(0.710)	(0.812)
Age	-0.00625**	-0.00388*	-0.000475	-0.00290	-0.00130	0.000452
	(0.019)	(0.078)	(0.834)	(0.334)	(0.592)	(0.858)
Woman	-0.314***	-0.236***	-0.215***	-0.323***	-0.212***	-0.285***
	(0.000)	(0.000)	(0.001)	(0.000)	(0.004)	(0.000)
Victim of robbery	-0.0726	0.0697	-0.218*	-0.0626	-0.138	-0.127
	(0.597)	(0.541)	(0.081)	(0.653)	(0.312)	(0.331)
Victim of theft	-0.248*	-0.129	-0.115	-0.156	0.0571	-0.0739
	(0.079)	(0.238)	(0.311)	(0.256)	(0.651)	(0.544)
Police satisfaction	0.0927**	0.119***	0.0939**	0.0872*	0.0992**	0.0904**
	(0.030)	(0.001)	(0.012)	(0.064)	(0.020)	(0.044)
Frequency of visits	-0.0800**	-0.0361	0.0270	-0.0773*	-0.0645*	-0.0263
	(0.025)	(0.221)	(0.377)	(0.058)	(0.056)	(0.446)
Education of income provider	0.0239	0.0454*	0.0317	0.00289	0.0180	-0.00393
	(0.369)	(0.066)	(0.202)	(0.927)	(0.482)	(0.882)
Income provider is working	-0.703	-0.0864	0.440	-1.011***	-0.427	-1.717***
	(0.145)	(0.845)	(0.617)	(0.004)	(0.181)	(0.000)
Income provider occupation FE	✓	✓	✓	✓	✓	✓
Sample size	516	513	512	515	514	515
R2	0.153	0.122	0.100	0.087	0.089	0.101

See details in the main text. Income provider refers to the main income provider of the respondent's household. Robust standard errors. p-values in parentheses. * p <0.1, ** p <0.05, *** p <0.01.

Table 4: Table 2 details, workers

	How safe in general			How safe at night		
	NH	SC	This SC	NH	SC	This SC
Treated x Post	-0.0341	0.116	0.0682	0.178	0.0229	0.506*
	(0.915)	(0.695)	(0.825)	(0.567)	(0.943)	(0.097)
Post intervention	0.0169	0.00847	-0.229	-0.0341	0.125	-0.383*
	(0.937)	(0.969)	(0.237)	(0.871)	(0.601)	(0.080)
Treated	0.0981	-0.0271	-0.332***	0.189	0.0268	-0.427***
	(0.526)	(0.839)	(0.007)	(0.212)	(0.837)	(0.000)
Age	0.00539	-0.00422	-0.0134***	0.00626	0.000487	-0.00592
	(0.367)	(0.445)	(0.010)	(0.286)	(0.924)	(0.224)
Woman	-0.366***	-0.178	-0.344***	-0.351***	-0.256**	-0.395***
	(0.005)	(0.115)	(0.001)	(0.007)	(0.023)	(0.000)
Victim of robbery	-0.123	-0.214	-0.128	-0.172	-0.164	-0.274*
	(0.506)	(0.216)	(0.352)	(0.334)	(0.297)	(0.057)
Victim of theft	-0.106	-0.120	-0.340**	-0.178	-0.181	-0.342**
	(0.582)	(0.499)	(0.032)	(0.318)	(0.281)	(0.027)
Police satisfaction	0.0272	0.0812*	0.0846**	0.0153	0.0521	0.0652
	(0.635)	(0.095)	(0.038)	(0.794)	(0.309)	(0.148)
Frequency of visits	-0.0774	0.0567	0.214***	-0.0241	0.0448	0.153
	(0.546)	(0.616)	(0.009)	(0.861)	(0.733)	(0.200)
Education of income provider	0.0227	0.00859	0.0519**	0.0131	-0.0181	0.0342
	(0.445)	(0.736)	(0.021)	(0.666)	(0.492)	(0.109)
Income provider is working	-0.315	-0.0546	-0.645**	0.240	0.536	-0.929***
	(0.463)	(0.853)	(0.034)	(0.612)	(0.137)	(0.001)
Income provider occupation FE	✓	✓	✓	✓	✓	✓
Sample size	253	253	251	252	254	252
R2	0.079	0.068	0.233	0.093	0.071	0.232

See details in the main text. Income provider refers to the main income provider of the respondent's household. Robust standard errors. p-values in parentheses. * p <0.1, ** p <0.05, *** p <0.01.

Table 5: Table 5 details, full sample

	How safe in general			How safe at night		
	NH	SC	This SC	NH	SC	This SC
Interacted with intervention	-0.289	-0.289	-0.476**	-0.284	-0.618***	-0.463**
	(0.195)	(0.195)	(0.018)	(0.220)	(0.004)	(0.048)
Age	-0.000114	-0.000114	0.00112	-0.00294	0.00421	0.00294
	(0.981)	(0.981)	(0.776)	(0.558)	(0.291)	(0.504)
Woman	-0.275**	-0.275**	-0.164	-0.365***	-0.364***	-0.342***
	(0.017)	(0.017)	(0.130)	(0.004)	(0.001)	(0.004)
Victim of robbery	-0.106	-0.106	-0.488*	-0.153	-0.181	-0.535*
	(0.739)	(0.739)	(0.084)	(0.631)	(0.542)	(0.070)
Victim of theft	-0.397	-0.397	-0.117	-0.379*	-0.0571	-0.126
	(0.108)	(0.108)	(0.538)	(0.084)	(0.796)	(0.539)
Police satisfaction	0.0419	0.0419	0.132**	0.0265	0.161**	0.104
	(0.562)	(0.562)	(0.047)	(0.755)	(0.030)	(0.179)
Frequency of visits	0.0538	0.0538	0.0368	-0.0704	-0.0179	-0.0408
	(0.372)	(0.372)	(0.512)	(0.303)	(0.732)	(0.491)
Education of income provider	-0.0524	-0.0524	-0.0106	-0.0113	-0.0218	-0.0444
	(0.211)	(0.211)	(0.752)	(0.811)	(0.587)	(0.224)
Income provider is working	-0.921*	-0.921*	0.641	0.253	0.407	-0.510
	(0.096)	(0.096)	(0.186)	(0.645)	(0.501)	(0.411)
Income provider occupation FE	✓	✓	✓	✓	✓	✓
Sample size	176	176	175	176	174	177
R2	0.141	0.141	0.156	0.094	0.184	0.173

See details in the main text. Income provider refers to the main income provider of the respondent's household. Robust standard errors. p-values in parentheses. * p <0.1, ** p <0.05, *** p <0.01.

Table 6: Table 5 details, non-workers

	How safe in general			How safe at night		
	NH	SC	This SC	NH	SC	This SC
Interacted with intervention	-0.0310	-0.451*	-0.247	-0.0943	-0.398*	-0.184
	(0.879)	(0.083)	(0.210)	(0.707)	(0.087)	(0.408)
Age	-0.00343	-0.00117	-0.000284	-0.00625	0.00304	0.00136
	(0.465)	(0.785)	(0.946)	(0.218)	(0.446)	(0.776)
Woman	-0.245**	-0.285**	-0.191*	-0.345***	-0.474***	-0.401***
	(0.038)	(0.023)	(0.096)	(0.010)	(0.000)	(0.002)
Victim of robbery	-0.320	-0.0981	-0.515	-0.237	-0.201	-0.599*
	(0.329)	(0.797)	(0.106)	(0.502)	(0.535)	(0.062)
Victim of theft	-0.396	-0.237	-0.173	-0.384	-0.0788	-0.147
	(0.128)	(0.268)	(0.398)	(0.103)	(0.749)	(0.518)
Police satisfaction	0.0473	0.0900	0.119	0.0386	0.186**	0.105
	(0.503)	(0.236)	(0.107)	(0.697)	(0.024)	(0.233)
Frequency of visits	-0.0489	-0.0441	-0.0403	-0.131*	-0.0922	-0.0834
	(0.436)	(0.498)	(0.519)	(0.087)	(0.116)	(0.224)
Education of income provider	-0.0455	0.0123	-0.0199	-0.0656	-0.0777*	-0.0626
	(0.256)	(0.799)	(0.626)	(0.224)	(0.078)	(0.202)
Income provider is working	-0.378	1.442***	1.195***	0.718*	1.087***	0.248
	(0.304)	(0.000)	(0.000)	(0.072)	(0.004)	(0.506)
Income provider occupation FE	✓	✓	✓	✓	✓	✓
Sample size	151	151	150	151	149	152
R2	0.159	0.136	0.159	0.134	0.237	0.186

See details in the main text. Income provider refers to the main income provider of the respondent's household. Robust standard errors. p-values in parentheses. * p <0.1, ** p <0.05, *** p <0.01.

Table 7: Table 5 details, workers

	How safe in general			How safe at night		
	NH	SC	This SC	NH	SC	This SC
Interacted with intervention	-1.455***	-1.413***	-1.552***	-1.272***	-1.784***	-1.689***
	(0.009)	(0.001)	(0.003)	(0.002)	(0.001)	(0.007)
Age	0.0190	0.0244*	0.0215	0.0313*	0.0226*	0.0279*
	(0.390)	(0.070)	(0.189)	(0.052)	(0.090)	(0.063)
Woman	-0.178	0.499	0.289	-0.0222	0.497	0.375
	(0.678)	(0.104)	(0.321)	(0.963)	(0.193)	(0.261)
Victim of robbery	0.405	-0.000212	-0.649	-0.146	-0.489	-0.218
	(0.585)	(1.000)	(0.275)	(0.836)	(0.285)	(0.742)
Victim of theft	-0.806	-0.236	0.152	-0.895	-0.348	-0.137
	(0.521)	(0.621)	(0.828)	(0.123)	(0.365)	(0.813)
Police satisfaction	0.00894	0.131	0.102	-0.236	-0.130	0.0629
	(0.973)	(0.526)	(0.716)	(0.237)	(0.463)	(0.750)
Frequency of visits	0.143	0.198	0.205	0.244	0.216	0.191
	(0.543)	(0.173)	(0.268)	(0.113)	(0.113)	(0.264)
Education of income provider	-0.0256	0.0820	0.00664	0.199*	0.166*	-0.00118
	(0.895)	(0.382)	(0.965)	(0.079)	(0.086)	(0.991)
Income provider occupation FE	✓	✓	✓	✓	✓	✓
Sample size	25	25	25	25	25	25
R2	0.416	0.516	0.383	0.608	0.573	0.555

See details in the main text. Income provider refers to the main income provider of the respondent's household. The dummy for the income provider working was omitted because it did not have variation. Robust standard errors. p-values in parentheses. * p <0.1, ** p <0.05, *** p <0.01.

Table 8: Table 6 details, full sample

	Difference-in-differences			Interacted with the intervention		
	Local gov.	Judiciary	Police	Local gov.	Judiciary	Police
Treated x Post	0.00898	-0.0557	-0.0700			
	(0.953)	(0.667)	(0.643)			
Post intervention	0.154	-0.0638	-0.00692			
	(0.208)	(0.527)	(0.953)			
Treated	-0.0728	0.111	0.124			
	(0.518)	(0.239)	(0.290)			
Interacted with intervention				0.00852	0.00279	0.198
				(0.973)	(0.991)	(0.373)
Age	-0.0117***	-0.00132	0.00405	-0.0137**	0.00384	0.00263
	(0.000)	(0.600)	(0.157)	(0.025)	(0.480)	(0.583)
Woman	0.0347	0.0309	0.159**	0.0617	0.119	0.328**
	(0.653)	(0.633)	(0.038)	(0.698)	(0.402)	(0.021)
Victim of robbery	-0.178	-0.195**	-0.239*	-0.211	-0.411	-0.708**
	(0.140)	(0.046)	(0.066)	(0.533)	(0.108)	(0.039)
Victim of theft	-0.0412	0.116	0.0794	-0.0571	-0.0321	0.0149
	(0.725)	(0.262)	(0.524)	(0.794)	(0.874)	(0.952)
Frequency of visits	0.0348	0.0110	-0.0257	-0.0210	-0.0148	-0.0184
	(0.308)	(0.698)	(0.457)	(0.814)	(0.830)	(0.794)
Education of income provider	0.0153	0.0585***	0.0676***	-0.0499	0.00240	0.0128
	(0.532)	(0.001)	(0.003)	(0.573)	(0.962)	(0.791)
Income provider is working	-0.533	-0.896	0.168	0.900***	1.043***	0.0300
	(0.319)	(0.175)	(0.730)	(0.010)	(0.001)	(0.958)
Income provider occupation FE	✓	✓	✓	✓	✓	✓
Sample size	661	751	774	154	171	178
R2	0.079	0.060	0.063	0.099	0.063	0.125

See details in the main text. Income provider refers to the main income provider of the respondent's household. Robust standard errors. p-values in parentheses. * p <0.1, ** p <0.05, *** p <0.01.

Table 9: Table 6 details, non-workers

	difference-in-differences			Interacted with the intervention		
	Local gov.	Judiciary	Police	Local gov.	Judiciary	Police
Treated x Post	-0.0553	-0.150	-0.126			
	(0.774)	(0.365)	(0.521)			
Post intervention	0.178	0.0331	0.0322			
	(0.231)	(0.799)	(0.840)			
Treated	-0.0416	0.163	0.180			
	(0.798)	(0.239)	(0.278)			
Interacted with intervention				0.0123	0.0850	0.295
				(0.965)	(0.766)	(0.206)
Age	-0.00962***	-0.000313	0.00243	-0.0111*	0.00304	0.00522
	(0.002)	(0.910)	(0.446)	(0.067)	(0.588)	(0.294)
Woman	0.0349	0.107	0.241***	0.137	0.109	0.339**
	(0.698)	(0.166)	(0.007)	(0.412)	(0.456)	(0.024)
Victim of robbery	-0.155	-0.0710	-0.210	-0.143	-0.499	-0.368
	(0.304)	(0.610)	(0.196)	(0.692)	(0.120)	(0.280)
Victim of theft	-0.176	0.0305	-0.0465	-0.272	-0.123	0.000778
	(0.227)	(0.827)	(0.769)	(0.230)	(0.603)	(0.997)
Frequency of visits	-0.0318	-0.0176	-0.0605	-0.118	-0.0937	-0.0580
	(0.482)	(0.646)	(0.169)	(0.266)	(0.230)	(0.482)
Education of income provider	-0.0262	0.00517	0.0308	-0.0953	-0.0539	-0.0565
	(0.445)	(0.845)	(0.374)	(0.303)	(0.376)	(0.335)
Income provider is working	-0.336	-1.718*	0.467	-0.473	1.420***	0.358
	(0.501)	(0.067)	(0.454)	(0.525)	(0.001)	(0.356)
Income provider occupation FE	✓	✓	✓	✓	✓	✓
Sample size	455	504	520	133	146	153
R2	0.075	0.050	0.073	0.090	0.112	0.150

See details in the main text. Income provider refers to the main income provider of the respondent's household. Robust standard errors. p-values in parentheses. * p <0.1, ** p <0.05, *** p <0.01.

Table 10: Table 6 details, workers

	Difference-in-differences			Interacted with the intervention		
	Local gov.	Judiciary	Police	Local gov.	Judiciary	Police
Treated x Post	0.513	0.255	0.0721			
	(0.214)	(0.465)	(0.841)			
Post intervention	-0.248	-0.366	-0.164			
	(0.410)	(0.169)	(0.533)			
Treated	-0.187	0.0442	0.0178			
	(0.250)	(0.759)	(0.918)			
Interacted with intervention				0.191	-0.259	-0.290
				(0.799)	(0.583)	(0.765)
Age	-0.0190***	-0.00225	0.00628	-0.0343	0.0117	-0.00711
	(0.002)	(0.717)	(0.358)	(0.134)	(0.540)	(0.639)
Woman	0.0301	-0.145	-0.0116	-0.633	0.237	-0.0698
	(0.837)	(0.222)	(0.937)	(0.305)	(0.630)	(0.869)
Victim of robbery	-0.191	-0.354**	-0.334	-0.935	-0.00704	-2.013***
	(0.356)	(0.015)	(0.123)	(0.217)	(0.989)	(0.001)
Victim of theft	0.105	0.199	0.264	1.036	0.490	-0.411
	(0.616)	(0.213)	(0.201)	(0.225)	(0.434)	(0.629)
Frequency of visits	0.188	0.213	0.0925	0.0324	0.108	-0.0401
	(0.153)	(0.171)	(0.580)	(0.855)	(0.676)	(0.790)
Education of income provider	0.0325	0.0942***	0.0863***	-0.115	0.0334	0.166
	(0.319)	(0.000)	(0.006)	(0.749)	(0.786)	(0.203)
Income provider is working	-1.219	-0.645	0.294	0	0	0
	(0.102)	(0.172)	(0.595)	(.)	(.)	(.)
Income provider occupation FE	✓	✓	✓	✓	✓	✓
Sample size	206	247	254	21	25	25
R2	0.105	0.110	0.080	0.480	0.087	0.424

See details in the main text. Income provider refers to the main income provider of the respondent's household. Robust standard errors. p-values in parentheses. * p <0.1, ** p <0.05, *** p <0.01.

Table 11: Table 7 details, full sample

	Victim	Victim near the shopping centre
Treated x Post	0.0390	-0.0428*
	(0.541)	(0.062)
Post intervention	-0.121**	0.0156
	(0.014)	(0.421)
Treated	-0.0213	0.0214
	(0.663)	(0.237)
Age	-0.00253**	-0.000244
	(0.026)	(0.493)
Woman	-0.0359	0.0126
	(0.263)	(0.234)
Police satisfaction	-0.0181	-0.00140
	(0.258)	(0.724)
Frequency of visits	-0.0152	-0.0113***
	(0.304)	(0.002)
Education of income provider	0.00948	0.00347
	(0.303)	(0.185)
Income provider is working	-0.181	-0.00330
	(0.461)	(0.835)
Income provider occupation FE	✓	✓
Sample size	786	786
R2	0.058	0.035

See details in the main text. Income provider refers to the main income provider of the respondent's household. Robust standard errors. p-values in parentheses. * p <0.1, ** p <0.05, *** p <0.01.

Table 12: Table 7 details, non-workers

	Victim	Victim near the shopping centre
Treated x Post	0.0275	-0.00616
	(0.747)	(0.862)
Post intervention	-0.0854	-0.0269
	(0.206)	(0.385)
Treated	0.00175	-0.0108
	(0.981)	(0.748)
Age	-0.00366***	-0.000648**
	(0.004)	(0.049)
Woman	-0.0167	0.0159
	(0.665)	(0.177)
Police satisfaction	-0.0219	-0.00371
	(0.286)	(0.467)
Frequency of visits	-0.0135	-0.0137**
	(0.481)	(0.021)
Education of income provider	-0.00303	0.00689**
	(0.835)	(0.039)
Income provider is working	0.0575	0.0207
	(0.898)	(0.431)
Income provider occupation FE	✓	✓
Sample size	529	529
R2	0.044	0.048

See details in the main text. Income provider refers to the main income provider of the respondent's household. Robust standard errors. p-values in parentheses. * p <0.1, ** p <0.05, *** p <0.01.

Table 13: Table 7 details, workers

	Victim	Victim near the shopping centre
Treated x Post	-0.00150	-0.0704
	(0.992)	(0.363)
Post intervention	-0.190*	0.0711
	(0.086)	(0.284)
Treated	-0.0459	0.0319
	(0.525)	(0.219)
Age	0.000810	0.000890
	(0.761)	(0.421)
Woman	-0.0593	0.00376
	(0.326)	(0.866)
Police satisfaction	-0.0237	0.00318
	(0.368)	(0.659)
Frequency of visits	-0.0518	-0.0155
	(0.321)	(0.179)
Education of income provider	0.0182	0.000505
	(0.177)	(0.893)
Income provider is working	0.147	0.00811
	(0.347)	(0.643)
Income provider occupation FE	✓	✓
Sample size	257	257
R2	0.095	0.056

See details in the main text. Income provider refers to the main income provider of the respondent's household. Robust standard errors. p-values in parentheses. * p <0.1, ** p <0.05, *** p <0.01.
