# Supplementary material for: The fear-increasing and fear-decreasing effects of a pilot policy to reduce fear of crime
Source: PLoS One. 2023 Mar 6;18(3):e0282461. doi: 10.1371/journal.pone.0282461 (PMC9987788; doi:10.1371/journal.pone.0282461)
Supplement: S2 Appendix — Extensions of Tables 4 and 5 with statistical tests for discrete variables. (PDF) [file pone.0282461.s004.pdf]

S2 Appendix

Table 14: **Differences in observable characteristics according to interaction with the intervention, full sample.**

	Average value		Sample size		p-value for significant difference		
**Variable**	**No interaction**	**Interaction**	**No interaction**	**Interaction**	**t-test**	**Mann-Whitney**	**Pearson's chi**
Woman	0.53	0.46	348	28	0.504	0.493	0.492
Victim of robbery (anywhere)	0.09	0.21	345	28	0.124	0.028	0.028
Victim of theft (anywhere)	0.11	0.14	342	28	0.592	0.538	0.538
Frequency of visits to this shopping centre	2.51	2.39	341	28	0.579	0.689	0.553
Education level of main income provider	9.31	9.68	347	28	0.235	0.607	0.757
Main income provider is employed	0.96	0.93	348	28	0.994	0.454	0.329
Type of job of main income provider	4.78	4.71	348	28	0.739	0.619	0.199
Police satisfaction (higher is better)	3.46	3.61	344	28	0.399	0.322	0.598

The table shows the estimated means and the respective sample sizes for the values of each variable for the individuals who *did* and *did not* interact with the intervention. The rightmost columns present p-values for statistical tests on whether the two groups are significantly different. For all tests, the null hypothesis is that the groups are not different. The *t-test* column presents the result for a two-sided t-test of differences in means, assuming unequal variances. The *Mann-Whitney* column for a Mann-Whitney-Wilcoxon test for independent samples. The *Pearson's chi* column for a Pearson's chi-squared test.

Table 15: **Differences in observable characteristics according to interaction with the intervention, non-workers.**

	Average value		Sample size		p-value for significant difference		
**Variable**	**No interaction**	**Interaction**	**No interaction**	**Interaction**	**t-test**	**Mann-Whitney**	**Pearson's chi**
Woman	0,51	0,46	304	24	0,615	0,606	0,605
Victim of robbery (anywhere)	0,08	0,21	301	24	0,148	0,034	0,033
Victim of theft (anywhere)	0,10	0,13	298	24	0,771	0,748	0,748
Frequency of visits to this shopping centre	2,70	2,63	297	24	0,729	0,937	0,505
Education level of main income provider	9.57	9.92	304	24	0.084	0.790	0.962
Main income provider is employed	0.95	0.92	304	24	0.532	0.415	0.414
Type of job of main income provider	4.91	4.88	304	24	0.858	0.597	0.370
Police satisfaction (higher is better)	3.50	3.67	300	24	0.374	0.265	0.501

The table shows the estimated means and the respective sample sizes for the values of each variable for the individuals who *did* and *did not* interact with the intervention. The rightmost columns present p-values for statistical tests on whether the two groups are significantly different. For all tests, the null hypothesis is that the groups are not different. The *t-test* column presents the result for a two-sided t-test of differences in means, assuming unequal variances. The *Mann-Whitney* column for a Mann-Whitney-Wilcoxon test for independent samples. The *Pearson's chi* column for a Pearson's chi-squared test.

Table 16: **Differences in observable characteristics according to interaction with the intervention, workers.**

	**Average value**		**Sample size**		**p-value for significant difference**		
**Variable**	**No interaction**	**Interaction**	**No interaction**	**Interaction**	**t-test**	**Mann-Whitney**	**Pearson's chi**
Woman	0,66	0,50	44	4	0,626	0,529	0,524
Victim of robbery (anywhere)	0,14	0,25	44	4	0,684	0,542	0,538
Victim of theft (anywhere)	0,11	0,25	44	4	0,627	0,435	0,430
Frequency of visits to this shopping centre	1,25	1,00	44	4	0,033	0,436	0,891
Education level of main income provider	7.47	8.25	43	4	0.690	0.458	0.573
Type of job of main income provider	3.89	3.75	44	4	0.844	1.000	0.457
Police satisfaction (higher is better)	3.18	3.25	44	4	0.900	0.953	0.853

The table shows the estimated means and the respective sample sizes for the values of each variable for the individuals who *did* and *did not* interact with the intervention. The rightmost columns present p-values for statistical tests on whether the two groups are significantly different. For all tests, the null hypothesis is that the groups are not different. The *t-test* column presents the result for a two-sided t-test of differences in means, assuming unequal variances. The *Mann-Whitney* column for a Mann-Whitney-Wilcoxon test for independent samples. The *Pearson's chi* column for a Pearson's chi-squared test. The variable '*Main income provider is employed*' is excluded because it has no within-group variation.
